# Multi-Dimensional Regression Models for Predicting the Wall Thickness Distribution of Corrugated Pipes

**DOI:** 10.3390/polym14173455

**Published:** 2022-08-24

**Authors:** Hanny Albrecht, Wolfgang Roland, Christian Fiebig, Gerald Roman Berger-Weber

**Affiliations:** 1 Pro2Future GmbH, Altenberger Strasse 69, 4040 Linz, Austria; 2 Institute of Polymer Processing and Digital Transformation, Johannes Kepler University Linz, Altenberger Strasse 69, 4040 Linz, Austria; 3 Unicor GmbH, Industrie Strasse 56, 97437 Hassfurt, Germany

**Keywords:** polymer processing, modeling and simulation, corrugated pipe, extrusion blow molding, symbolic regression

## Abstract

Corrugated pipes offer both higher stiffness and higher flexibility while simultaneously requiring less material than rigid pipes. Production rates of corrugated pipes have therefore increased significantly in recent years. Due to rising commodity prices, pipe manufacturers have been driven to produce corrugated pipes of high quality with reduced material input. To the best of our knowledge, corrugated pipe geometry and wall thickness distribution significantly influence pipe properties. Essential factors in optimizing wall thickness distribution include adaptation of the mold block geometry and structure optimization. To achieve these goals, a conventional approach would typically require numerous iterations over various pipe geometries, several mold block geometries, and then fabrication of pipes to be tested experimentally—an approach which is very time-consuming and costly. To address this issue, we developed multi-dimensional mathematical models that predict the wall thickness distribution in corrugated pipes as functions of the mold geometry by using symbolic regression based on genetic programming (GP). First, the blow molding problem was transformed into a dimensionless representation. Then, a screening study was performed to identify the most significant influencing parameters, which were subsequently varied within wide ranges as a basis for a comprehensive, numerically driven parametric design study. The data set obtained was used as input for data-driven modeling to derive novel regression models for predicting wall thickness distribution. Finally, model accuracy was confirmed by means of an error analysis that evaluated various statistical metrics. With our models, wall thickness distribution can now be predicted and subsequently used for structural analysis, thus enabling digital mold block design and optimizing the wall thickness distribution.

## 1. Introduction

**Figure 1:**
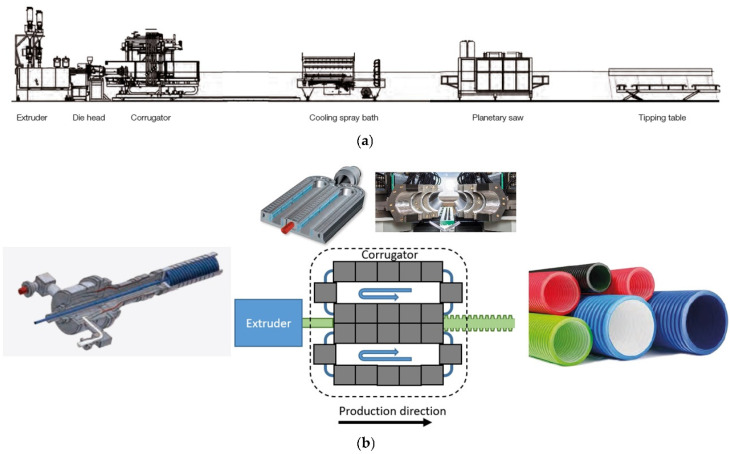
(**a**) Schematic of a corrugated pipe production line and of the corrugator with (**b**) the die head.

Extrusion blow molding (EBM) is a manufacturing process in which thermoplastics are molten inside the extruder, and then the molten polymer is extruded through an annular die head forming a hollow tube called a parison. The parison is subsequently captured by cooled mold blocks, inflated into the mold cavity by pressurization (blowing air and/or vacuum suction) and blown into its final desired shape. Inside the molds, the corrugated pipe is then cooled down and solidified before it exits the corrugator. If necessary, further active cooling can be applied before it is cut or rolled up. A schematic of the corrugated pipe production line is depicted in Figure 1. Production of corrugated pipes is a typical example of extrusion blow molding. The overall extrusion process resembles classic pipe extrusion, with the difference that the extrudate is inflated and formed into its final shape inside the corrugator. Forming of the pipe can be supported and optimized by using a vacuum in the mold blocks [1].

Corrugated pipe technology is well-established and pipes can be made from a large variety of material types, such as high-density polyethylene (HDPE), polypropylene (PP), polyamide (PA), polyvinylchloride (PVC), and thermoplastic elastomers (TPE). Compared with rigid and non-corrugated pipes, corrugated pipes offer higher flexibility and are therefore more versatile and used in a wide range of applications, such as cable protection and technical applications in the automotive industry, machine construction, healthcare, telecommunication, households, land and road drainage, and sewerage and storm water disposals. The available diameter range is from approximately 3 mm to 2400 mm. Other advantages of corrugated pipes are high production speed due to low weight, material economy due to specific structural design, easy handling, resistance to corrosion, high ring stiffness compared with rigid pipes of the same weight, and excellent water flow due to the smooth surface of the inner layer of double-wall corrugated pipes [1].

Particularly important for the performance of the pipes are wall thickness distribution, pipe weight, and mechanical properties. A careful balance of these three parameters must be maintained during the pipe manufacturing process, as they are highly interdependent. The wall thickness distribution is a key factor in pipe design because it significantly impacts the final quality of the pipe and the production costs. For example, thick pipe walls offer additional strength, but during the cooling phase they tend to warp more and require increased cycle time, and their weight increases material cost. 

In recent years, many researchers have sought to predict the wall thickness of the final blown part. Thibault et al. [2] developed a predictive preform geometry software and optimal operating conditions for the stretch blow molding process. This numerical approach uses a constrained gradient-based algorithm that iterates automatically over finite element software to optimize the operating conditions. In thermoforming, Rosenzweig et al. [3] developed a theoretical isothermal one-dimensional geometric model that predicts wall-thickness profiles of vacuum- or pressure-formed products. The model is based on some simplifying assumptions and is independent of material properties and forming conditions.

In extrusion blow molding, Debbaut et al. [4] carried out viscoelastic blow molding simulations using a realistic viscoelastic constitutive model of the integral type. They concentrated on the thickness distribution of the blown product and used a fluid membrane element in a Lagrangian formulation combined with an efficient contact algorithm. With this approach, they were able to numerically simulate the blow molding process of an industrial part with a relatively complex geometry. Further research [5,6,7,8,9,10,11,12] using computer simulations, with a focus mainly on optimization of the wall thickness distribution, has been conducted in extrusion blow molding.

At this point, only a few wall thickness prediction models are available, especially for corrugated pipes. Hence, we investigated the impact of mold geometry parameters on the wall thickness distribution and developed a geometry-dependent wall thickness model. All relevant independent geometry parameters were investigated and transformed into dimensionless form by applying the theory of similarity and dimensional analysis. A comprehensive parametrically driven finite element method (FEM) design study of the blow-molding process was conducted for a wide parameter range. Based on these results, approximation equations were derived by means of symbolic regression using genetic programming. The accuracy of our models was evaluated using error analysis. The models were further optimized, simplified, and also validated with an independent dataset that was not previously considered in the modeling.

This hybrid approach was recently applied by our research group to investigate the flow in metering sections of single-screw extruders [13,14,15,16,17], the flow of polymer melts through melt filtration systems [18,19], and co-extrusion die flows [20]. The results showed that the integrated approach is well suited to solving complex problems in the area of polymer processing. Moreover, the created mathematical models are well suited for manufacturers as a smart tool for predicting the wall thickness distribution and the wall thickness ratio of corrugated pipes.

## 2. Fundamentals

### 2.1. Geometry and Modeling

**Figure 2:**
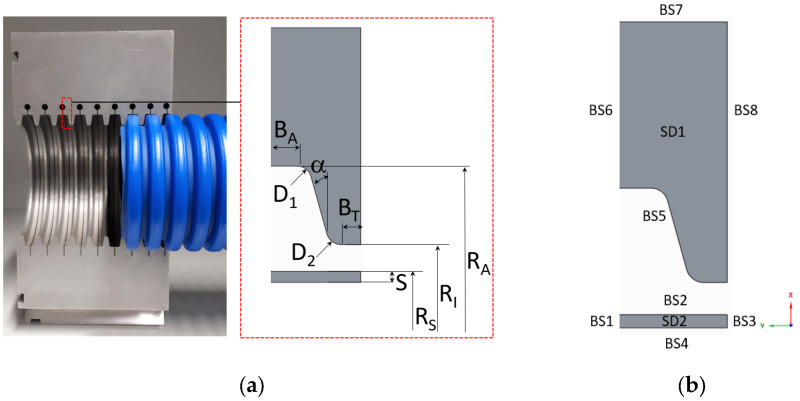
(**a**) 3D Mold block geometry on which the 2D axisymmetric model is based; (**b**) simulation boundary conditions.

The geometry of the mold block considered in this study is given in Figure 2a. The complex 3D model was simplified to obtain a 2D axisymmetric model to significantly reduce modeling and analysis time. Due to the symmetry of the geometry, it is sufficient to model only half the period of the corrugated pipe geometry. Furthermore, a single-wall corrugated pipe is considered. Figure 2 shows all geometry parameters considered for parameterization: mold block inner radius ($R_{I}$), mold block outer radius ($R_{A}$), crest diameter ($D_{1}$), valley diameter ($D_{2}$), half-profile width at crest ($B_{A}$), half-profile width at valley ($B_{T}$), initial thickness of the extruded fluid parison (S), initial outer radius of the fluid parison ($R_{S}$), and flank angle (α). As illustrated in Figure 2, the problem domain is divided into two sub-domains (SD) and seven boundaries (BS). Subdomain 1 (SD1) and subdomain 2 (SD2) represent the geometry of fluid parison and mold block, respectively. The boundary conditions applied to the problem are as follows:

- BS1 and BS3: symmetry
- BS2: free surface, constant normal forces (vacuum pressure) imposed, contact detection problem with the solid mold defined
- BS4: free surface, constant normal forces (inflation air pressure) imposed
- BS5: contact wall (contact between fluid parison and wall)
- BS6, BS7 and BS8: no contact walls and free of force

The following assumptions were applied in this study:

- The thickness of the extruded fluid parison, S, is constant in the initial state before the blow molding process.
- The temperature of the extruded fluid parison, S, is homogeneous and thus the viscosity remains constant over the entire cross section.
- The influence of the viscoelasticity, temperature, and pressures (air and vacuum) was neglected as they are insignificant for the final wall thickness distribution. However, they have an impact on the dynamics of the process, i.e., on how fast the molding process takes place.
- The extrusion speed of the parison is exactly equal to the speed of the mold blocks. Based on this assumption, it is allowed to neglect the dynamics of the process and reduce the geometry to a half model.

### 2.2. Dimensional Analysis and Similitude

The Buckingham ∏ theorem and the theory of similarity [21] are applied for constructing dimensionless parameters and ensuring geometric similarity in the problem. Dimensional analysis is extremely useful for several reasons: it reduces the number of influencing parameters needed to describe the problem and identifies the characteristic independent influencing parameters. This considerably simplifies the parametric design study that follows. Furthermore, generalized results are obtained in dimensionless representation, being valid for any dimensional representation covered by the dimensionless space by applying scaling rules.

Due to the assumptions made in the previous chapter, we were therefore able to focus only on the geometric similarity in the problem and avoid having too many Π terms in the final solution. The mold geometry in the problem was designed such that it can be arbitrarily modified. The outer radius of the mold block ($R_{A}$) was selected as the scaling parameter for defining the dimensionless parameters and therefore remained fixed. 

Eight independent dimensionless parameters identified as influencing factors are generated using the following equations by scaling them with $R_{A}$ (except for α).

(1) $$\prod_{n}=\frac{n}{R_{A}}\;;\;\;n=R_{I},\;B_{T},\;B_{A},\;D_{1},\;D_{2},\;S,\;R_{S}.$$

(2) α=α.

**Figure 3:**
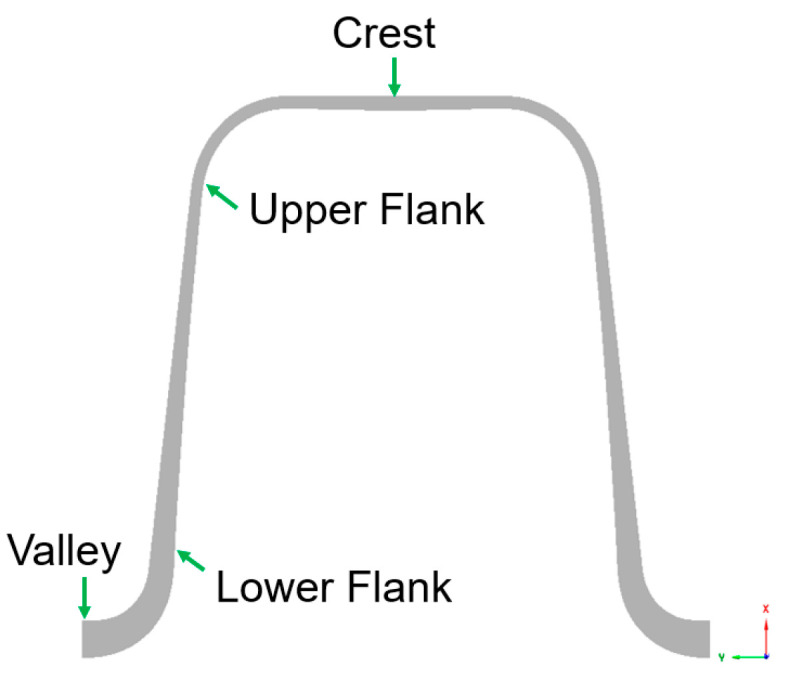
Wall thickness evaluation positions: crest, valley, lower and upper flank.

The target parameters in this analysis are the dimensionless wall thickness (Equation (3)) evaluated at one of the four representative positions ($\prod_{TP\_i}$), as shown in Figure 3, and the ratio of the wall thickness at the crest to the wall thickness at the valley (Equation (4)).

(3) $$\prod_{TP\_i}=\frac{TP\_i}{R_{A}}\;;\;\;i=Crest\;\left(C\right),\;Valley\left(V\right),\;Lower\;Flank\;\left(LF\right),\;and\;Upper\;Flank\;\left(UF\right).$$

(4) $$Ratio=\frac{\prod_{TP\_C}}{\prod_{TP\_V}}$$

As dimensionless influencing parameters, the dimensionless fluid parison thickness $\prod_{S}$ and the dimensionless parison initial outer radius $\prod_{R_{s}}$ are identified. However, as long as the tube is blown up freely—this means it is not in contact with the mold—the parison thickness decreases while the parison radius increases by maintaining its volume, respectively cross-sectional area. Hence, these two parameters are not independent and the dimensionless parison outer radius $\prod_{R_{s}}$ will be kept constant at $\prod_{R_{S*}}$, and the dimensionless initial parison thickness $\prod_{S}$ is selected as the independent influencing parameter. A dimensional problem that is governed by a dimensionless parison initial outer radius $\prod_{R_{s}}$ that is different than the defined $\prod_{R_{S*}}$, can also be described by a dimensionless representation with $\prod_{R_{S*}}$ by transforming the dimensionless initial fluid parison thickness $\prod_{S}$ to $\prod_{S*}$ according to Equation (5), which is based on volume conservation:

(5) $$\prod_{S_{*}}=\prod_{R_{S_{*}}}+\sqrt{\prod_{R_{s}{}_{*}}-2\prod_{R_{s}}\prod_{S}+\prod_{S}{}^{2}}$$

## 3. Numerical Simulation

**Figure 4:**
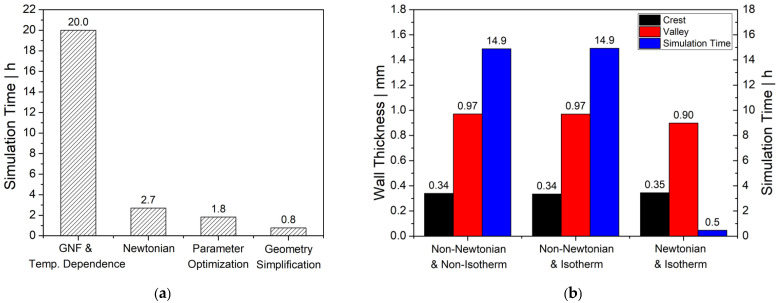
(**a**) Optimization of the simulation time, (**b**) comparison study of wall thickness distribution and simulation time.

In this work, a time-dependent and isothermal parison inflation process of an incompressible Newtonian fluid was simulated using the commercial FEM-based computational fluid dynamics (CFD) software package Ansys Polyflow [22]. Since—as previously mentioned—shear plays a minor role during parison inflation, the Newtonian model was chosen. Viscoelasticity can be omitted because the elastic properties will not influence the final wall thickness distribution. However, it will have an impact on the dynamics of the blow molding process, and hence on the shaping time. Our initial preliminary study, conducted on a regular working notebook with Intel(R) Core (TM) i7-8550U CPU @ 1.80GHz processor and 32 GB RAM, also indicated that simplifying the rheological modeling approach from generalized Newtonian fluid (GNF) to Newtonian fluid model was able to significantly reduce the computational time by 86.5% (see Figure 4a). Further parameter optimization and geometry simplification could also optimize the simulation time by 32.2% and 57.5%, respectively, without sacrificing accuracy of the wall thickness distribution (see Figure 4b). 

**Figure 5:**
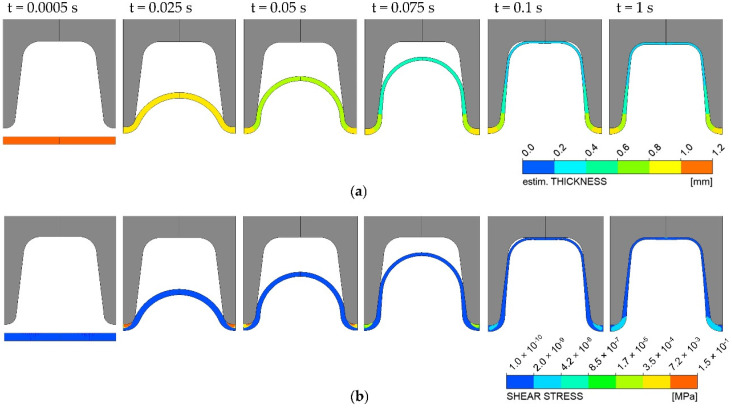
Simulation results of the blow-molding process showing that the parison is fully inflated after an inflation time of 1 s: (**a**) wall thickness distribution, (**b**) shear stress distribution.

Since simulation software works only in dimensional representation, an equivalent dimensional setup has to be constructed for each dimensionless parameter. For this purpose, a mold block geometry for a medium-sized corrugated pipe with an outer diameter of 200 mm ($R_{A}$ = 102.95 mm) was selected as a reference. The molten parison was inflated inside the mold block and assumed to have a constant viscosity of 22,000 Pa.s and a melt density $\rho_{m}$ of 728.5 kg/m^3^. For operational conditions, a constant vacuum and inflation air pressure of 0.9 and 0.1 bar were applied, respectively, to the outer and inner surfaces of the fluid parison. This non-linear problem was solved numerically and iteratively by a very robust algebraic multi-frontal (AMF) direct solver based on the Gauss elimination method [22]. The final converged solution was obtained after performing the time-dependent calculation with the assigned parameters needed by the iterative scheme. Subsequently, the results are transformed back into a dimensionless representation. The blowing process over time is exemplarily shown in Figure 5 for various time steps. It can be seen that at first, the parison is inflated uniformly until it gets in contact with the mold. Then, the parison is further inflated into the mold, next getting into contact with the flanks and subsequently with the crest. The upper flank radius is shaped last, after an inflation time of approximately 1 s.

**Figure 6:**
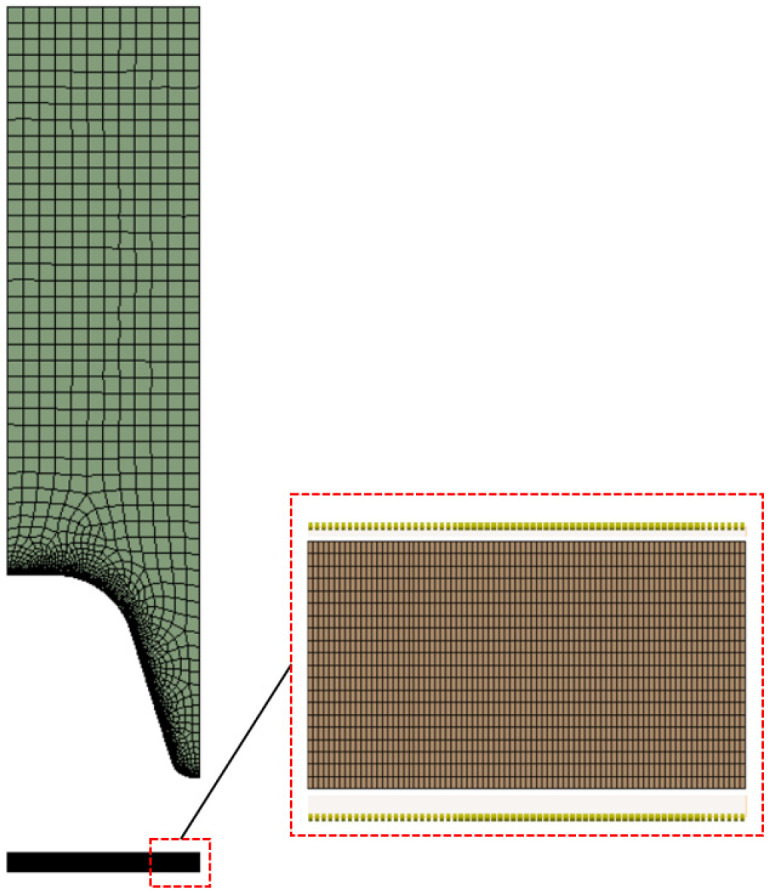
Finite element meshes used.

Prior to the parametric design study, a mesh-independence study was also performed on an HP Z800 workstation with dual core Xeon processors and 48 GB RAM to determine a mesh for the simulation that creates optimal solutions, which are independent of the mesh resolution, as well as provide simulation results within reasonable times. In general, the finer the mesh, the more accurate the solution and the longer the computational time. For this analysis, as illustrated in Figure 6, a mold block and a fluid parison geometry were discretized into a set of two-dimensional hexahedral and wedge mesh elements. Meshes were generated by varying the edge size in the fluid parison subdomain within a specified range. Subsequently, preliminary simulations were performed in order to investigate significant differences in wall thickness that are due to mesh resolution. Since the fractional factorial parametric design study involved a large dataset with various geometry configurations, we sought to minimize the number of elements generated for each mesh to keep the simulation time as short as possible while ensuring that sufficiently accurate results could be achieved. Table 1 shows the estimated wall thickness at some evaluation points for various meshes.

The wall thicknesses estimated based on the various meshes were almost identical. There were differences of only 0.6% at the crest and of less than 0.2% at the valley between the results from the finest and coarsest meshes. Since computational time was also a crucial factor in conducting the fractional factorial parametric design study, the mesh with an edge size of 0.075 mm in fluid parison length was chosen to balance speed and accuracy. As the simulation was parameterized completely in terms of mold geometry, solving of the numerical problem of the fluid parison inflation process was automatically driven by the simulation solver. Since the number of time steps might differ in each new calculation, we considered only results from the selected upper time limit (end of inflation time at 1 s). 

## 4. Design Study

### 4.1. Screening Design

First, the dimensionless influencing parameters which significantly influence the wall thickness distribution of the corrugated pipe are identified by a screening design study. To this end, a statistical screening design of experiments (DoE) with center and star points that represent the low and high values of the factors, respectively, was selected. A screening design generally involves only a small number of experimental runs and is therefore more efficient and less costly than a corresponding full-factorial design. A center point was included in the design to increase efficiency and determine the curvature of the model.

#### 4.1.1. Screening Design—Procedure

In the screening design, the reference geometry was set as the center point of the multi-dimensional design space. All seven independent geometry parameters were selected as factors, and three levels were considered for each factor (see Table 2). For this analysis, the wall thickness ratio (Equation (4)) was chosen as the target parameter as it indirectly represents two wall thicknesses. The numerical results from this simulation study were then exported and re-written in dimensionless form for further analysis.

To determine the significance of each individual influencing parameter, the simulation output data were statistically analyzed. The probability value (*p*-value), which measures the strength of the evidence against the null hypothesis, was chosen to identify those geometry parameters that had a significant influence on the wall thickness distribution ratio and those that could be ignored. 

In this study, we followed the general *p*-value approach that has been widely adopted in practice. If the *p*-value is less than the specified significance level (α=0.05, which is usually used in technical applications), the null hypothesis *H_0_* is rejected, and it can be concluded that the difference is significant. In other words, with a *p*-value < 0.05, the result is statistically significant, and with a *p*-value > 0.05, it is not [23].

Multiple linear regression was used to capture the functional relationships between dependent (wall thickness ratio) and independent parameters (all influencing geometry parameters). The estimated regression equation is given by (Equation (6)):

(6) $$Ratio=\beta_{0}+\beta_{1}\cdot \prod_{R_{I}}+\beta_{2}\cdot \prod_{D_{1}}+\beta_{3}\cdot \prod_{D_{2}}+\beta_{4}\cdot \prod_{B_{A}}+\beta_{5}\cdot \prod_{B_{T}}+\beta_{6}\cdot \prod_{S}+\beta_{7}\cdot \alpha ,$$

where $\beta_{0},\;\;\beta_{1},\;\ldots \;\beta_{7}$ are the regression coefficients. The first step in performing a test of statistical significance is to define the null-hypothesis and alternative-hypothesis. The constant $\beta_{0}$ is not tested. The null-hypothesis for the other individual coefficient states that the coefficient is zero (Equation (7)), and the alternative-hypothesis that the coefficient is non-zero (Equation (8)).

(7) $$H_{0,i}:\beta_{i}=0,\;i=1\ldots 7;$$

(8) $$H_{A,i}:\beta_{i}\neq \;0,\;i=1\ldots 7.$$

In order to test our hypotheses, the simulation output data were collected, and the *t*-test for checking the significance (*p*-value) of individual regression coefficients in the multiple linear regression model was computed.

#### 4.1.2. Screening Design—Results 

**Figure 7:**
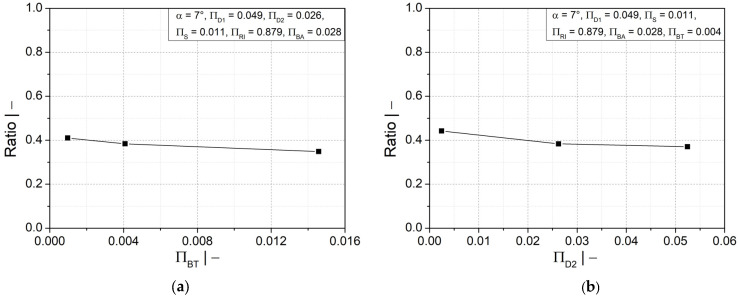
Ratio of wall thicknesses at crest and valley as a function of dimensionless (**a**) half profile width at valley $\prod_{B_{T}}$ and (**b**) valley diameter $\prod_{D_{2}}$.

**Figure 8:**
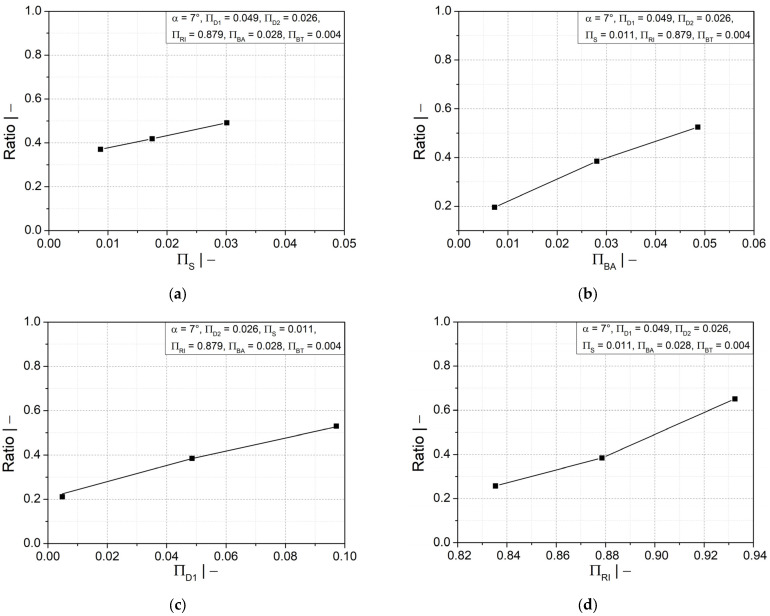

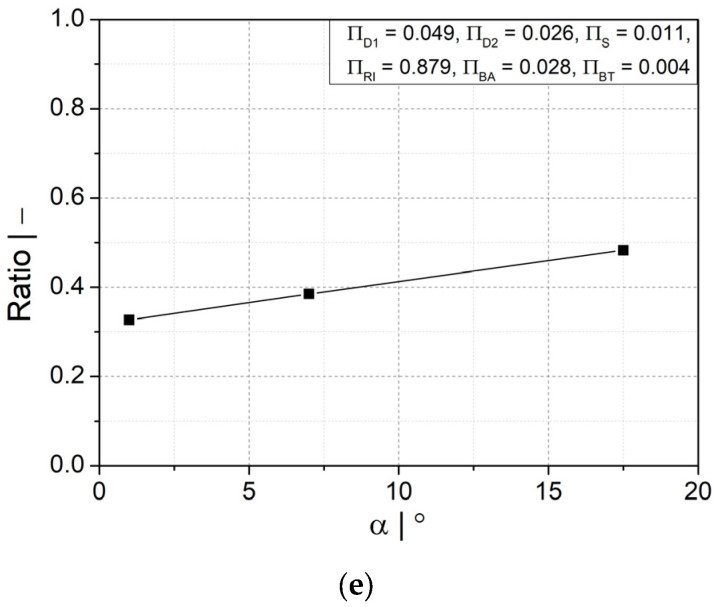
Ratio of wall thicknesses at crest and valley as a function of dimensionless (**a**) initial thickness of fluid parison $\prod_{S};\;($**b**) half profile width at crest $\prod_{B_{A}}$; (**c**) crest diameter $\prod_{D_{1}}$; (**d**) mold block inner radius $\prod_{R_{I}}$; and (**e**) flank angle α.

The results of the screening design study revealed that the profile width at valley ($\prod_{B_{T}}$) and valley diameter ($\prod_{D_{2}}$) (see Figure 7a,b) had little influence on the wall thickness distribution compared to the other geometry parameters (see Figure 8a–e). In addition, the results of multiple regression analysis of the screening design study, Table 3, also confirmed the previous finding. Based on the usual *p*-value cutoff of 0.05, only five geometry parameters ($\prod_{R_{I}}$, $\prod_{D_{1}}$, $\prod_{B_{A}}$, $\prod_{S}$, and α) were found to be statistically significant, and the other two ($\prod_{B_{T}}$ and $\prod_{D_{2}}$) were insignificant and were therefore discarded.

### 4.2. Parametric Design Study

After the screening design study, a five-level parametric design study was conducted by varying the selected independent geometry parameters as listed in Table 4. The selected parameter range covers a very wide range of available mold-block geometries and pipe dimensions commonly used in industrial corrugated pipe manufacturing. 

#### Parametric Design Study—Results

**Figure 9:**
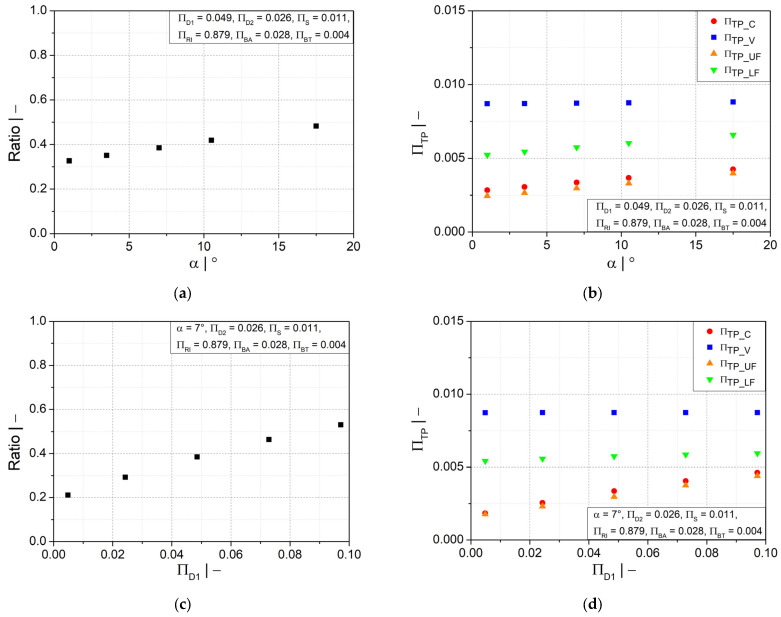
Ratio of wall thicknesses at crest and valley and dimensionless wall thickness as functions of (**a**,**b**) flank angle α and (**c**,**d**) dimensionless crest diameter $\prod_{D_{1}}$.

Figure 9a–d shows that increasing the flank angle and the crest diameter will lead to an increase in the wall thickness ratio and its distribution, specifically in the crest and upper flank areas. A small positive influence was also found in the lower flank area due to larger flank angles, but less impact due to crest diameter. In the valley area, neither geometry parameter had an impact. A larger flank angle has the advantage of allowing a smooth gradual change in wall thickness. 

**Figure 10:**
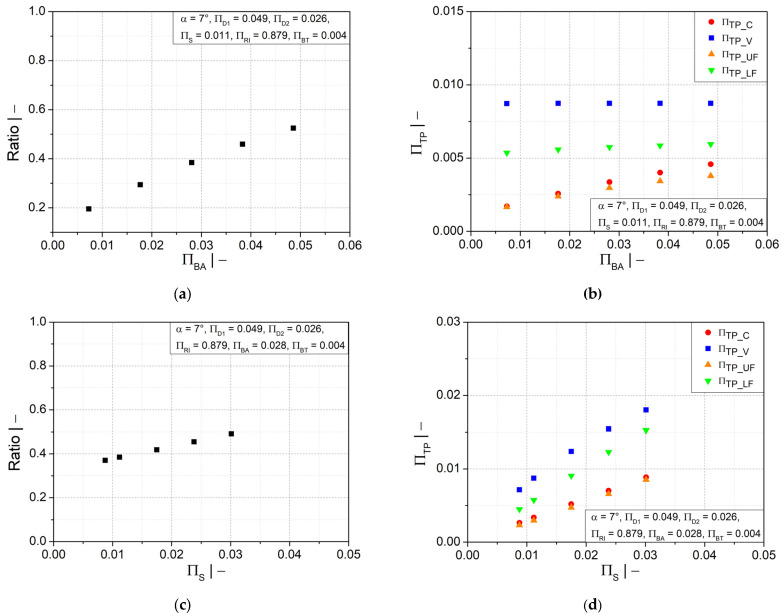
Ratio of wall thicknesses at crest and valley and dimensionless wall thickness as functions of dimensionless (**a**,**b**) half profile width at crest $\prod_{B_{A}}$ and (**c**,**d**) initial thickness of fluid parison $\prod_{S}$.

A more homogeneous wall thickness distribution between the crest and valley could also be obtained by a larger profile width at the crest, as illustrated in Figure 10a. However, as depicted in Figure 10b, a significant increase in wall thickness is observed only at the crest and upper flank, while the increase is less significant at the lower flank area and insignificant at the valley area. Figure 10c shows that the initial fluid parison thickness has a similar influence on the wall thickness distribution as the flank angle. A thicker initial fluid parison ensures an increase in wall thickness at all positions, as shown in Figure 10d. However, a reasonable final weight of the pipe must be considered to save material. 

**Figure 11:**
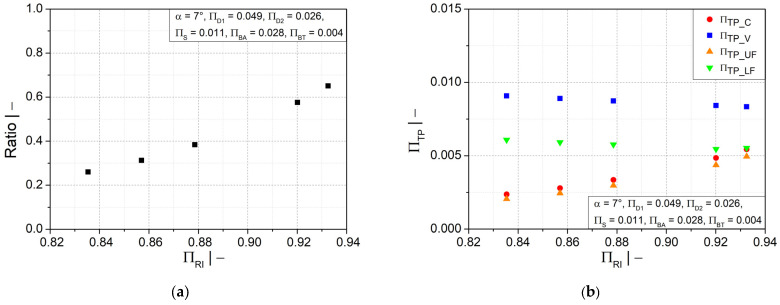
(**a**) Ratio of wall thicknesses at crest and valley and (**b**) dimensionless wall thickness as functions of dimensionless mold block inner radius $\prod_{R_{I}}$.

Additionally, the ratio of the wall thicknesses at the crest and at the valley is improved by a larger mold inner radius, as shown in Figure 11a; the distance between crest and valley was reduced to avoid a large variation in wall thickness. Figure 11b shows that increasing the mold inner radius would also increase the wall thickness in the crest and in the upper flank area, but the wall thickness in the valley and the lower flank would decrease.

## 5. Regression Analysis Using Heuristic Approaches

The previous sections presented the results of the numerically driven parametric design study revealing the relationships between the dimensionless influencing parameters of the mold geometry and the wall thickness distribution of the corrugated pipe. An analytical expression for the wall thickness distribution as a function of the dimensionless influencing parameters is still missing. In order to avoid numerical simulations for further analysis and enable prediction of the wall thickness distribution for any arbitrary combination of the defined influencing parameters within the defined parameter space, multi-dimensional mathematical models that describe wall-thickness distribution and ratio as functions of the influencing mold geometry parameters were developed. These models can save development time and reduce cost, resulting in industry-wide benefits. In addition, our analytical models enable more efficient modeling and exploration of new rational and practical corrugated pipe designs since the target values of new processes can be accurately predicted even without product manufacturing. In addition, the models allow direct interpolation between the data in multi-dimensional space since the simulations are only the discrete points in space and the regression is a hypersurface.

### 5.1. Symbolic Regression—Modeling

In order to derive symbolic regression models that best describe the relationship between the defined target parameters (wall thickness at thickness points) and of the identified independent input parameters for corrugated pipes, heuristic approaches based on genetic programming (GP) were employed. In this study, we used HeuristicLab [24], an open-source software system for heuristic optimization that offers several metaheuristic optimization algorithms and addresses several optimization problems.

In principle, symbolic regression is a data-based methodology the goal of which is to discover functions that best describe a given dataset with minimal error [25]. Unlike conventional linear or nonlinear regression methods that require a specific model structure and pre-defined parameters, symbolic regression based on genetic algorithms searches over the space of all possible formulas and their coefficients defined by a set of user-specified mathematical objects that are arithmetic operators (+, −, *, /, etc.), mathematical functions (trigonometric, exponential, logarithmic, etc.), and terminal sets (constant and independent variable). 

**Figure 12:**
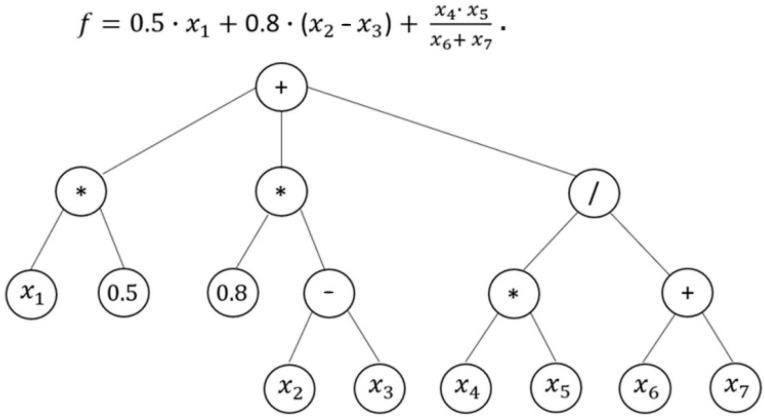
Symbolic regression model in mathematical notation and in the form of a parse tree.

The use of GP evolves in the process for implementing the symbolic regression, and the formulas are expressed as parse trees (see Figure 12). GP starts with the generation/creation of a random initial population of individuals (i.e., mathematical models), and then computes the fitness of each individual in this population. A new individual population of models is created by using crossover and mutation until the stop criterion is met, and at the end of the process, the output is the fittest model so far [26]. Throughout this evolutionary process, variables that have more impact will also be identified and become significant. This methodology has the advantages that the models generated are, firstly, able to express nonlinear relationships that are more complex than those of conventional regression methods, and secondly, can be interpreted and inspected by domain experts [27]. Furthermore, the predicted model is a mathematical expression and can thus be transformed, manipulated, and easily incorporated into expert systems [24].

The emphasis in this work was on generating mathematical models that predict wall thickness distribution and ratio in corrugated pipes. These models should also be applicable in practice to a broad range of materials and a wide range of geometries. The derived models allow us to gain further insight into how changing certain factors (influencing geometry parameters) affects the response behavior (wall thickness distribution and ratio). 

In our study, a variant of single-objective symbolic regression using an offspring-selection genetic algorithm (OSGA) [28] was employed. In order to speed up the solving process, only about two thirds of the full dataset (2083 design points)—collected randomly from numerical simulation results—was loaded into HeuristicLab. Since the model was to be trained on training data and then applied to the test dataset, the input dataset was split before we set up other parameters. In our implementation, the modeling dataset partition was 66% for training and 34% for testing to assess the performance of our model. Furthermore, some constraints on mathematical building blocks were set by limiting the functions to be used in the predictions such that the search space of all possible equations in symbolic regression was also limited and computational time reduced. Table 5 shows the optimized parameter settings that were used with the heuristic algorithm after several preliminary experiments. To account for statistical variations in the initial population, experiments were run in 20 independent repetitions with the same parameter setting until the solution converged to the best derived models. 

### 5.2. Symbolic Regression—Results

The symbolic regression equations derived for the wall thickness ratio and for the dimensionless wall thickness at crest and valley are:

(9) $$Ratio={\left(A_{1}+\frac{A_{2}}{\left(A_{3}+A_{4}+A_{5}+\frac{\left(A_{6}+{\left(A_{7}+\frac{A_{8}\;A_{9}}{A_{10}}\right)}^{2}\right)}{A_{11}}\right)}\right)}^{2}A_{12}+A_{13}.$$

(10) $$\prod_{TP\_C}=B_{1}+\frac{B_{2\;}(\left(B_{3}+B_{4}\;\left(B_{5}\;B_{6}+B_{7}\right){)}^{2}+B_{8}\right)}{B_{9}}.$$

(11) $$\prod_{TP\_V}=\left(C_{1}\;C_{2}\;C_{3}+C_{4}\right)\;C_{5}{\left(\frac{C_{6}}{C_{7}}\right)}^{2}C_{8}+C_{9}.$$

Similar models were also derived for the dimensionless wall thickness in the lower and upper flanks.

(12) $$\prod_{TP\_LF}=E_{1}+E_{2}\left(E_{3}+E_{4}+E_{5}\right)+E_{6}+E_{7}.$$

(13) $$\prod_{TP\_UF}=F_{1}+\frac{F_{2}\;F_{3}}{F_{4}}+{\left(\frac{F_{5}}{F_{6}}\right)}^{2}F_{7}.$$

Here, $A_{1}-A_{13}$, $B_{1}-B_{9}$, $C_{1}-C_{9}$, $E_{1}-E_{7}$, and $F_{1}-F_{7}$ in Equations (9)–(13) are sub-functions that contain some constants $a_{00}-a_{38},b_{00}-b_{27},\;c_{00}-c_{32},\;e_{00}-e_{32},$ and $\;f_{00}-f_{29}$ (see Appendix A).

**Figure 13:**
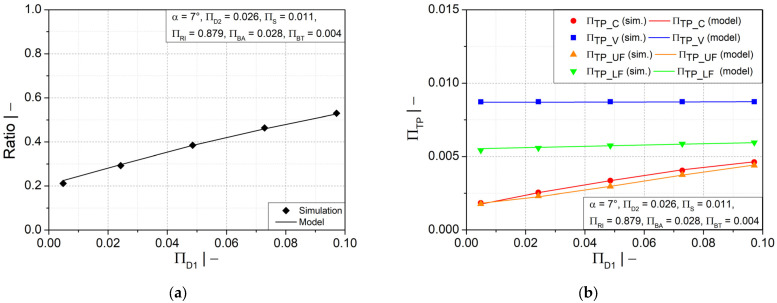

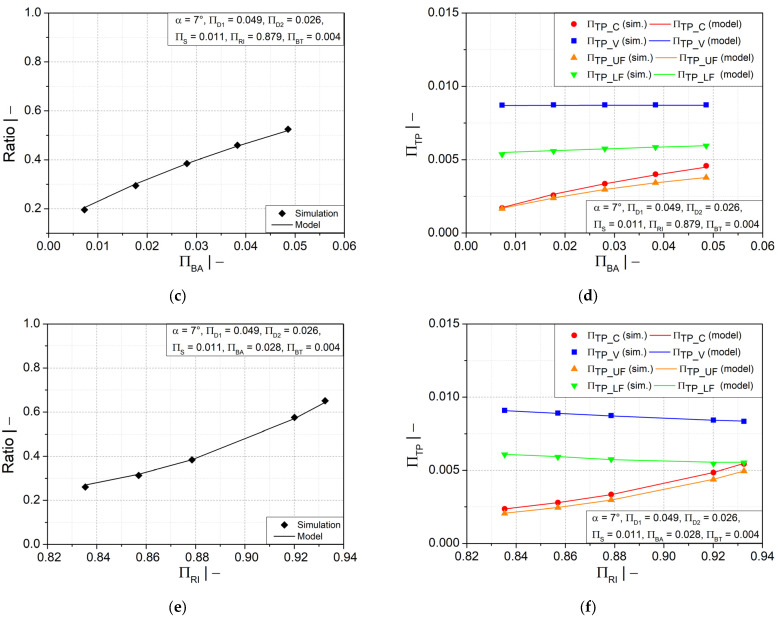
Comparisons of the estimated models for wall thickness distribution ratio and dimensionless wall thickness at several evaluation positions as functions of (**a**,**b**) dimensionless crest diameter $\prod_{D_{1}}$, (**c**,**d**) dimensionless half profile width at crest $\prod_{B_{A}},\;$ (**e**,**f**) dimensionless mold block inner radius $\prod_{R_{I}}$.

**Figure 14:**
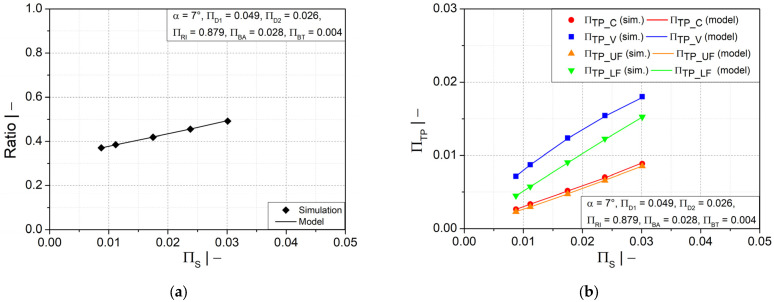

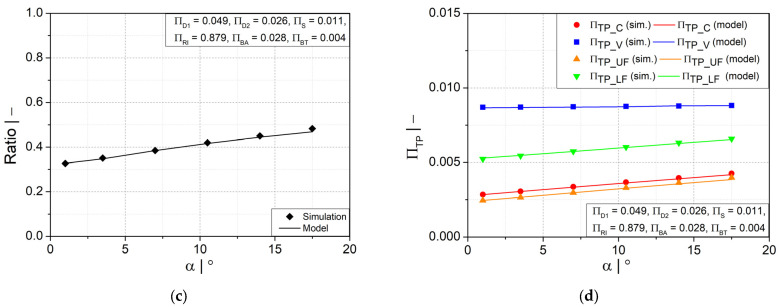
Comparisons of the estimated models for wall thickness distribution ratio and dimensionless wall thickness at several evaluation positions as functions of (**a**,**b**) dimensionless initial thickness of fluid parison $\prod_{S}$ and (**c**,**d**) flank angle α.

Comparisons of all symbolic regression models with the numerical simulations results are presented in Figure 13a–f for the most significant influencing parameters ($\prod_{R_{I}}$, $\prod_{D_{1}}\;$ and $\prod_{B_{A}}$), and in Figure 14a–d for two other geometry parameters ($\prod_{S}$ and *α*). As can be seen, the models are in excellent agreement with the simulation results.

To determine how our models perform on new data, we additionally evaluated them on previously unseen validation datasets (not used in model training) comprising 273 design points that were randomly chosen from Table 6.

Afterwards, the global accuracy of the derived models was also evaluated on validation data prior to the practical application by performing some error analyses. The coefficient of determination—Pearson *R*^2^ (Equation (14))—which describes how close the values estimated by our models are to the measured values, and is indicative of the response variation explained by a model, was utilized to determine the model quality.

(14) $$R^{2}=1-\frac{\sum_{i=1}^{N}{\left(y_{i}-{\hat{y}}_{i}\right)}^{2}}{\sum_{i=1}^{N}{\left(y_{i}-{\bar{y}}_{i}\right)}^{2}}.$$

where N is the number of observations, $y_{i}$ is the real value for observation i, ${\hat{y}}_{i}$ is the predicted value of y for observation i, and $\;{\bar{y}}_{i}$ is the mean value of the real value. A high *R*^2^ value (=1) indicates that the predictions agree perfectly with the measured values, and a low *R*^2^ value (=0) means that the predictions are poor and that the model is to be discarded [26]. 

In addition, the mean absolute error (MAE) (Equation (15)) and the mean relative error (MRE) (Equation (16)) were also analyzed for the derived models.

(15) $$\mathrm{MAE}=\frac{\;1}{N}\sum_{i=1}^{N}\left|y_{i}-{\hat{y}}_{i}\right|.$$

(16) $$\mathrm{MRE}=\frac{\;1}{N}\sum_{i=1}^{N}\frac{\left|y_{i}-{\hat{y}}_{i}\right|}{y_{i}}.$$

**Figure 15:**
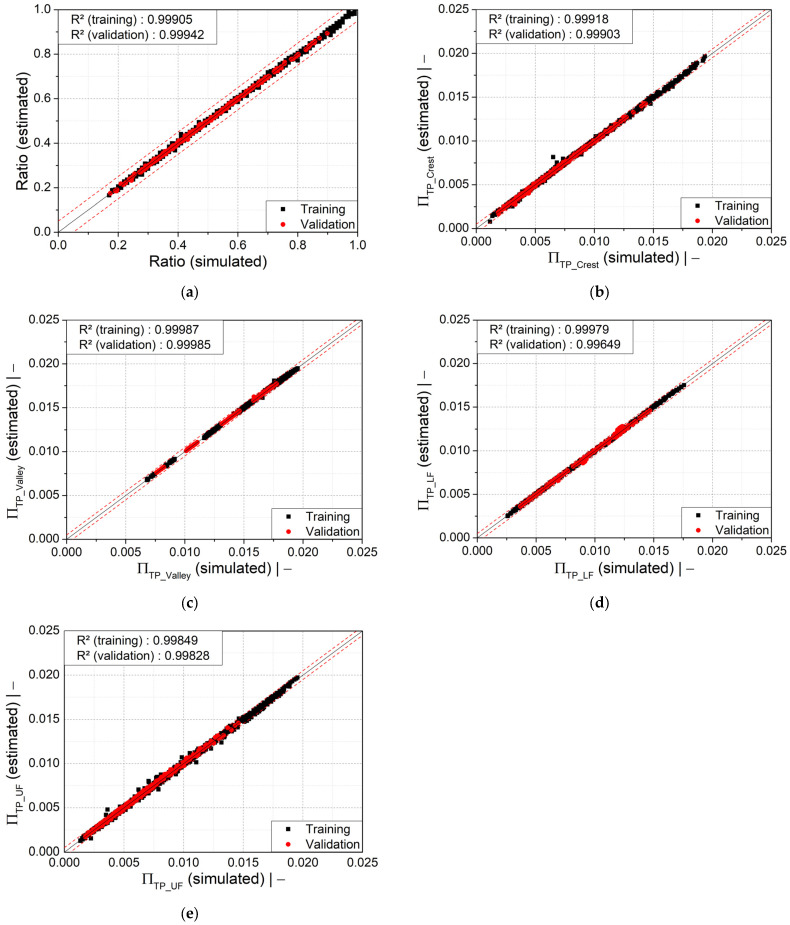
Scatter plots of (**a**) wall thickness ratio, (**b**) dimensionless wall thickness at the crest, (**c**) dimensionless wall thickness at the valley, (**d**) dimensionless wall thickness at the lower flank, and (**e**) dimensionless wall thickness at the upper flank. The dashed red lines indicate a relative error of ±5%.

The scatter plots in Figure 15a–e illustrate that most of the values calculated using the regression models (Equations (9)–(13) accord well with the numerically obtained simulation results. Most of the data points are within the range of maximum relative error of ±5%. Some outliers visible in Figure 15b,e are due to numerical errors and non-converged solutions, but their influence on the model is minimal. 

Generally, it can be concluded that the predictive models are in very good agreement, as a high coefficient of determination R^2^ was achieved after final model evaluation. Subsequently, the statistical accuracies of all derived models based on validation data set are given in Table 7. As indicated, the models given by Equations (9)–(13) achieved relatively small MAE values and MRE ≤ 1.632%, as well as the coefficient of determination R^2^ > 0.996. The error analyses thus confirm that the derived models are statistically highly accurate. 

## 6. Conclusions

Multi-dimensional regression models of the wall thickness distribution as a function of mold geometries in extrusion blow molding of corrugated pipes were developed using heuristic approaches. The influences of major geometry parameters on the parison inflation process were identified and investigated by applying the theory of similarity, dimensional analysis, and a parametric design study. Screening design results revealed that the impacts of valley profile width and diameter on wall thickness distribution were relatively small compared with those of other mold geometry parameters. This was confirmed by a null hypothesis test. Three independent geometry parameters (mold inner radius, profile width, and diameter at crest) were identified as having the most significant influences on the wall thickness distribution; two further independent geometry parameters (flank angle and initial thickness of the fluid parison) were identified as having a less significant positive influence on wall thickness distribution. The correlations between independent and target parameters can be established and utilized to estimate the wall thickness and its distribution in corrugated pipes. Moreover, the comparison of numerical simulation results and model predictions also confirmed the validity and feasibility of the regression models developed in this work. Considering the identified correlation between geometry and wall thickness distribution, these models are also suitable for optimizing mold blocks and pipes: high-quality pipes can thus be constructed using less time and material. First comparisons with experimental trials delivered promising results. These results showed that the wall thickness predictions capture the reality as long as the velocity of the extruded parison approximately equals the line speed of the corrugator. Currently, further experiments are planned for subsequent validation.

For new processes, the proposed method may prove to be a valuable tool for minimizing the number of expensive and time-consuming experiments when evaluating (new) pipe designs and may add value well before the final product is produced. The developed models allow a target variable (of the corrugated pipe geometry) to be predicted without manufacturing and prototyping of a product. In addition, the regression models can cover a wide range of geometry variations as they are dimensionless, and as long as the new geometry is within the chosen dimensionless geometry parameter range. For very small and very large corrugated pipes, there is some risk that the dimensionless parameters fall within the extrapolation range for various reasons. 

## Figures and Tables

**Table 1:** Mesh sensitivity analysis.

Parameter	Mesh 1	Mesh 2	Mesh 3	Mesh 4	Mesh 5	Mesh 6
Edge Sizes [mm]	0.01	0.025	0.05	0.075	0.1	0.15
CPU Time [s]	5282	2300	1358	992	953	519
Wall thickness at crest [mm]	0.3475	0.3470	0.3450	0.3447	0.3435	0.3417
Wall thickness at valley [mm]	0.8997	0.8992	0.8992	0.8987	0.8985	0.8976

**Table 2:** Range of geometry parameters in the screening design.

Parameter	Unit	Value		
$\prod_{R_{I}}$	-	0.835	0.879	0.932
$\prod_{D_{1}}$	-	0.005	0.049	0.097
$\prod_{D_{2}}$	-	0.002	0.026	0.052
$\prod_{B_{A}}$	-	0.007	0.028	0.049
$\prod_{B_{T}}$	-	0.001	0.004	0.015
$\prod_{S}$	-	0.009	0.011	0.030
α		1	7	17.5

**Table 3:** Multiple regression analysis output from the screening design study.

Term	Estimated Regression Coefficient	*p*-Value
$\prod_{R_{I}}$	0.0401725	6.25418 × 10^−5^
$\prod_{D_{1}}$	0.0332355	0.000293
$\prod_{D_{2}}$	−0.013532	0.186159
$\prod_{B_{A}}$	0.0775136	0.000227
$\prod_{B_{T}}$	−0.040225	0.226997
$\prod_{S}$	0.0526789	0.020054
α	0.0092651	0.012899

**Table 4:** Geometry parameter ranges for the parametric design study.

Parameter	Unit			Value		
$\prod_{R_{I}}$	-	0.835	0.857	0.879	0.920	0.932
$\prod_{D_{1}}$	-	0.005	0.024	0.049	0.073	0.097
$\prod_{B_{A}}$	-	0.007	0.018	0.028	0.038	0.049
$\prod_{S}$	-	0.009	0.011	0.017	0.024	0.030
α		1	3.5	7	10.5	17.5

**Table 5:** OSGA parameter configurations.

Parameter	Value
Population size	100
Selected parents	200
Crossover probability	90%
Mutation probability	25%
Maximum tree depth	30
Maximum tree length	100
Fitness function	Pearson *R^2^*
Maximum generations	75
Maximum selection pressure	100
Operators	+, −, ∗, /
	Power functions (square)

**Table 6:** Geometry parameter ranges for the validation dataset.

Parameter	Unit		Value		
$\prod_{R_{I}}$	-	0.846	0.868	0.899	0.926
$\prod_{D_{1}}$	-	0.015	0.036	0.061	0.085
$\prod_{B_{A}}$	-	0.012	0.023	0.033	0.043
$\prod_{S}$	-	0.010	0.014	0.021	0.027
α	°	2.25	5.25	8.75	14

**Table 7:** Results of the error analysis of the wall thickness prediction models.

Model	R^2^ (−)	MAE (−)	MRE (%)
Ratio	0.99942	3.101 × 10^−3^	0.720
$\prod_{TP\_Crest}$	0.99903	6.255 × 10^−5^	1.232
$\prod_{TP\_Valley}$	0.99985	2.550 × 10^−5^	0.194
$\prod_{TP\_LF}$	0.99649	1.058 × 10^−4^	1.073
$\prod_{TP\_UF}$	0.99828	9.235 × 10^−5^	1.632

## Data Availability

The data presented in this study are available on request from the corresponding author.

## Appendix A

### Appendix A.1. Sub-Functions for the Regression model Predicting Ratio

(A1)
$$A_{1}=a_{00}\;\pi_{S}+a_{01}\;\pi_{D_{1}}+a_{02}\;\alpha +a_{03}\;\pi_{B_{A}}+a_{36}.$$

(A2)
$$A_{2}=\frac{1}{a_{04}\;\pi_{R_{I}}}+a_{05}.$$

(A3)
$$A_{3}=a_{06}\;\pi_{S}+a_{07}\;\pi_{R_{I}}+a_{08}\;\pi_{B_{A}}+a_{09}\;\pi_{D_{1}}+a_{10}\;\alpha .$$

(A4)
$$A_{4}={\left(a_{11}\;\pi_{S}+a_{12}\;\pi_{B_{A}}+\frac{a_{13}\;\alpha }{\pi_{R_{I}}}+\frac{1}{a_{14}\;\pi_{R_{I}}}+a_{15}\right)}^{2}.$$

(A5)
$$A_{5}=\pi_{D_{1}}{\left(a_{16}\;\pi_{D_{1}}+a_{17}\pi_{B_{A}}\right)}^{2}+a_{35}.$$

(A6)
$$A_{6}=a_{18}\;\alpha +a_{19}\;\pi_{B_{A}}+a_{20}\;\pi_{D_{1}}.$$

(A7)
$$A_{7}=a_{21}\;\pi_{R_{I}}+\frac{1}{a_{33}\;\pi_{R_{I}}}.$$

(A8)
$$A_{8}=a_{22}\;\pi_{D_{1}}+a_{23}\;\pi_{S}+\frac{a_{24}\;\alpha }{\pi_{R_{I}}\;\pi_{R_{I}}}.$$

(A9)
$$A_{9}={\left(a_{25}\;\alpha +\frac{\left(a_{26}\;\pi_{D_{1}}+a_{27}\;\pi_{S}\right)}{\left(a_{28}\;\pi_{D_{1}}+a_{29}\;\pi_{B_{A}}\right)}+\pi_{R_{I}}\;\alpha \;a_{30}\right)}^{2}.$$

(A10)
$$A_{10}=a_{31}\;\pi_{D_{1}}+a_{32}\;\pi_{B_{A}}.$$

(A11)
$$A_{11}=a_{34}\;\pi_{R_{I}}.$$

(A12)
$$A_{12}=a_{37},\;\;A_{13}=a_{38}.$$

**Table A1:** Rounded values of the constant for Equation (1).

Constant	Value	Constant	Value	Constant	Value	Constant	Value
$a_{00}$	5.102	$a_{10}$	−7.786 × 10^−2^	$a_{20}$	5.052	$a_{30}$	−2.894 × 10^−1^
$a_{01}$	−3.763 × 10^−1^	$a_{11}$	5.775	$a_{21}$	1.388	$a_{31}$	6.817
$a_{02}$	−1.967 × 10^−3^	$a_{12}$	−7.779	$a_{22}$	4.833	$a_{32}$	1.197
$a_{03}$	−1.588	$a_{13}$	−1.202 × 10^−2^	$a_{23}$	1.754	$a_{33}$	−6.157 × 10^−1^
$a_{04}$	−1.909 × 10^−1^	$a_{14}$	−1.103	$a_{24}$	−3.799 × 10^−3^	$a_{34}$	6.009
$a_{05}$	5.199	$a_{15}$	6.869 × 10^−1^	$a_{25}$	2.320 × 10^−1^	$a_{35}$	8.257 × 10^−1^
$a_{06}$	8.229	$a_{16}$	3.796	$a_{26}$	−3.956 × 10^−1^	$a_{36}$	1.114
$a_{07}$	−8.324 × 10^−1^	$a_{17}$	1.562 × 10^1^	$a_{27}$	4.595	$a_{37}$	8.906 × 10^−1^
$a_{08}$	7.659	$a_{18}$	4.302 × 10^−1^	$a_{28}$	1.890	$a_{38}$	1.002 × 10^−1^
$a_{09}$	5.134	$a_{19}$	−7.264	$a_{29}$	5.028		

### Appendix A.2. Sub-Functions for the Regression Model Predicting $\prod_{TP\_C}$

(A13)
$$B_{1}={\left(b_{00}\;\pi_{S}+b_{01}\;\pi_{R_{I}}+b_{02}\;\pi_{B_{A}}+b_{03}\;\alpha +\frac{1}{b_{04}\;\pi_{S}}\right)}^{2}+b_{27}.$$

(A14)
$$B_{2}=\pi_{s}\;b_{20}.$$

(A15)
$$B_{3}=b_{05}\;\pi_{R_{I}}+\pi_{D_{1}}\left(b_{06}\;\pi_{D_{1}}+b_{07}\right).$$

(A16)
$$B_{4}=b_{08}\;\alpha +b_{09}.$$

(A17)
$$B_{5}=\pi_{B_{A}}\;\pi_{S}\;\left(b_{10}\;\alpha +b_{11}\;\pi_{S}+b_{12}\right).$$

(A18)
$$B_{6}={\left(\frac{\left(b_{13}\;\pi_{B_{A}}+b_{14}\;\pi_{S}\right)}{{\left(\frac{b_{15}\;\pi_{B_{A}}+b_{16}\;\pi_{D_{1}}}{\pi_{S}}\right)}^{2}}\right)}^{2}+b_{17}.$$

(A19)
$$B_{7}=b_{18},\;\;B_{8}=b_{19}.$$

**Table A2:** Rounded values of the constant for Equation (2).

Constant	Value	Constant	Value	Constant	Value	Constant	Value
$b_{00}$	2.283	$b_{07}$	−1.010 × 10^−1^	$b_{14}$	1.874	$b_{21}$	8.775 × 10^−2^
$b_{01}$	7.534 × 10^−2^	$b_{08}$	5.358 × 10^−4^	$b_{15}$	7.546 × 10^−2^	$b_{22}$	4.176 × 10^−2^
$b_{02}$	1.808 × 10^−1^	$b_{09}$	−0.112	$b_{16}$	3.625 × 10^−2^	$b_{23}$	1.293 × 10^−4^
$b_{03}$	4.289 × 10^−4^	$b_{10}$	−1.602 × 10^−1^	$b_{17}$	−1.189 × 10	$b_{24}$	−3.388 × 10^−2^
$b_{04}$	−1.917 × 10^3^	$b_{11}$	−2.469 × 10^−1^	$b_{18}$	2.060	$b_{25}$	−1.225 × 10^−1^
$b_{05}$	−8.858 × 10^−1^	$b_{12}$	2.468	$b_{19}$	−1.189	$b_{26}$	31.48
$b_{06}$	−8.320 × 10^−1^	$b_{13}$	−1.094	$b_{20}$	1.762 × 10^2^	$b_{27}$	4.664 × 10^−3^

### Appendix A.3. Sub-Functions for the Regression Model Predicting $\prod_{TP\_V}$

(A20)
$$C_{1}=\left(c_{0}\;\pi_{R_{I}}+c_{1}\;\pi_{B_{A}}\right).$$

(A21)
$$C_{2}=\left(c_{2}\;\alpha +{\left(c_{3}\;\pi_{R_{I}}+c_{4}\right)}^{2}\;{\left(c_{5}\;\pi_{R_{I}}+c_{6}\right)}^{2}\left(c_{7}\;\pi_{B_{A}}+\pi_{S}\;\pi_{D_{1}}c_{8}\right)\;\left(c_{9}\;\alpha +c_{10}\right)+c_{11}\right).$$

(A22)
$$C_{3}=\left(c_{12}\;\pi_{S}+c_{13}\;\pi_{R_{I}}\right).$$

(A23)
$$C_{4}=c_{14}$$

(A24)
$$C_{5}=\left(c_{15}\;\pi_{S}+c_{16}\;\pi_{R_{I}}\right).$$

(A25)
$$C_{6}=\left(c_{17}\;\pi_{D_{1}}+c_{18}\right).$$

(A26)
$$C_{7}=\left(c_{19}\;\pi_{R_{I}}+\pi_{S}\;\pi_{R_{I}}\;c_{20}+\left(c_{21}\;\pi_{B_{A}}+\pi_{B_{A}}\;\pi_{D_{1}}\;c_{22}\right){\left({\left(c_{23}\;\pi_{R_{I}}\right)}^{2}+c_{24}\right)}^{2}+c_{25}\right).$$

(A27)
$$C_{8}=\left(c_{26}\;\alpha +c_{27}\;\pi_{D_{1}}+c_{28}\;\pi_{R_{I}}+\frac{c_{29}\;\pi_{R_{I}}}{\pi_{S}}+c_{30}\right)c_{31}.$$

(A28)
$$C_{9}=c_{32}.$$

**Table A3:** Rounded values of the constant for Equation (3).

Constant	Value	Constant	Value	Constant	Value	Constant	Value
$c_{00}$	1.355	$c_{09}$	−2.382 × 10^−1^	$c_{18}$	−21.15	$c_{27}$	−10.48
$c_{01}$	−1.756	$c_{10}$	11.28	$c_{19}$	1.566 × 10^2^	$c_{28}$	−19.62
$c_{02}$	−7.893 × 10^−2^	$c_{11}$	11.22	$c_{20}$	−1.540 × 10^2^	$c_{29}$	3.222
$c_{03}$	17.11	$c_{12}$	1.925	$c_{21}$	1.750 × 10^−2^	$c_{30}$	−2.579 × 10^2^
$c_{04}$	−11.62	$c_{13}$	1.002 × 10^−1^	$c_{22}$	3.369 × 10^−1^	$c_{31}$	1.004
$c_{05}$	17.19	$c_{14}$	−3.621	$c_{23}$	10.13	$c_{32}$	8.134 × 10^−3^
$c_{06}$	−11.62	$c_{15}$	1.739	$c_{24}$	17.24		
$c_{07}$	−7.048 × 10^−3^	$c_{16}$	2.974 × 10^−3^	$c_{25}$	8.078 × 10^2^		
$c_{08}$	−1.687 × 10^−1^	$c_{17}$	−2.246	$c_{26}$	−2.228 × 10^−1^		

### Appendix A.4. Sub-Functions for the Regression Model Predicting $\prod_{TP\_LF}$

(A29)
$$E_{1}=e_{00}\;\pi_{S}.$$

(A30)
$$E_{2}=\pi_{S}\;e_{12}.$$

(A31)
$$E_{3}=e_{01}\;\alpha +e_{02}\;\pi_{D_{1}}+e_{03}\;\pi_{B_{A}}+e_{04}\;\pi_{S}.$$

(A32)
$$E_{4}={\left(e_{05}\;\alpha +\frac{e_{06}\;\pi_{D_{1}}}{\pi_{S}}+e_{07}\right)}^{2}.$$

(A33)
$$E_{5}=\pi_{B_{A}}{}^{2}\;e_{08}+{\left(e_{09}\;\alpha +e_{10}\;\pi_{D_{1}}+e_{11}\;\pi_{S}\right)}^{2}.$$

(A34)
$$\begin{array}{c} \;E_{6}=(e_{13}\;\pi_{S}+e_{14}\;\pi_{D_{1}}+e_{15}\;\pi_{R_{I}}\\ +(e_{16}\;\alpha +e_{17}\;\pi_{D_{1}}+e_{18}\;\pi_{B_{A}}+\pi_{S}\;\pi_{R_{I}}\;e_{19}+e_{20})\;(e_{21}\;\alpha +e_{22}\;\pi_{S}\\ +e_{23}\;\pi_{B_{A}}+e_{24}\;\pi_{D_{1}}+e_{25}\;)+e_{26}\;{)}^{2}e_{27}. \end{array}$$

(A35)
$$E_{7}={\left(e_{28}\;\pi_{S}+e_{29}\;\pi_{D_{1}}+e_{30}\;\pi_{R_{I}}+e_{31}\right)}^{2}+e_{32}.$$

**Table A4:** Rounded values of the constant for Equation (4).

Constant	Value	Constant	Value	Constant	Value
$e_{00}$	4.114 × 10^−1^	$e_{11}$	1.461	$e_{22}$	2.473
$e_{01}$	6.584 × 10^−3^	$e_{12}$	1.219	$e_{23}$	1.736
$e_{02}$	3.588 × 10^−2^	$e_{13}$	8.923 × 10^−1^	$e_{24}$	7.159 × 10^−1^
$e_{03}$	9.938 × 10^−1^	$e_{14}$	−1.334 × 10^−1^	$e_{25}$	−1.772 × 10^−1^
$e_{04}$	−2.344	$e_{15}$	−8.782 × 10^−1^	$e_{26}$	7.645 × 10^−1^
$e_{05}$	−5.395 × 10^−3^	$e_{16}$	2.443 × 10^−3^	$e_{27}$	−1.009
$e_{06}$	−1.518 × 10^−2^	$e_{17}$	1.046	$e_{28}$	−1.245
$e_{07}$	1.876 × 10^−1^	$e_{18}$	1.730	$e_{29}$	−1.074 × 10^−1^
$e_{08}$	−3.506	$e_{19}$	−10.680	$e_{30}$	−1.016
$e_{09}$	−2.727 × 10^−3^	$e_{20}$	−1.563 × 10^−1^	$e_{31}$	8.869 × 10^−1^
$e_{10}$	8.860 × 10^−1^	$e_{21}$	5.067 × 10^−3^	$e_{32}$	−1.991 × 10^−4^

### Appendix A.5. Sub-Functions for the Regression Model Predicting $\prod_{TP\_UF}$

(A36)
$$F_{1}=\left(f_{00}\;\pi_{R_{I}}+f_{01}\;\pi_{D_{1}}+f_{02}\;\pi_{S}\right)+f_{29}.$$

(A37)
$$F_{2}=\pi_{S}\left(f_{03}\;\alpha +f_{04}\;\pi_{R_{I}}+f_{05}\;\pi_{D_{1}}+\pi_{B_{A}}\;\pi_{R_{I}}\left(\pi_{B_{A}}\;\pi_{R_{I}}\;f_{06}+f_{07}\right)+\frac{fi_{08}}{\pi_{D_{1}}}+\alpha \;{\left(\pi_{R_{I}}\right)}^{2}\;f_{09}+{\left({\left(f_{10}\;\pi_{R_{I}}\right)}^{2}+f_{11}\right)}^{2}\right).$$

(A38)
$$F_{3}=\left(f_{12}\;\pi_{S}+f_{13}\;\pi_{R_{I}}+f_{14}\;\alpha +\pi_{B_{A}}\;\left(\pi_{B_{A}}\;\pi_{R_{I}}\;f_{15}+f_{16}\right)\right).$$

(A39)
$$F_{4}=f_{17}\;\pi_{R_{I}}+\frac{f_{18}}{\pi_{D_{1}}}.$$

(A40)
$$F_{5}=\pi_{S}\left(f_{19\;}\pi_{R_{I}}+f_{20}\;\pi_{S}+\alpha \;{\left({\left(f_{21}\;\pi_{R_{I}}\right)}^{2}+f_{22}\right)}^{2}f_{23}+{\left(f_{24}\;\pi_{R_{I}}\right)}^{2}\right).$$

(A41)
$$F_{6}=f_{25}\;\pi_{R_{I}}+f_{26}\;\pi_{B_{A}}+f_{27}\;\pi_{D_{1}}.$$

(A42)
$$F_{7}=f_{28}.$$

**Table A5:** Rounded values of the constant for Equation (5).

Constant	Value	Constant	Value	Constant	Value
$f_{00}$	−4.410 × 10^−3^	$f_{11}$	−1.41418	$f_{22}$	−1.925
$f_{01}$	−2.280 × 10^−3^	$f_{12}$	2.249	$f_{23}$	−2.795 × 10^−2^
$f_{02}$	−3.333	$f_{13}$	4.547	$f_{24}$	1.276
$f_{03}$	9.931 × 10^−3^	$f_{14}$	1.510 × 10^−2^	$f_{25}$	2.415 × 10^−2^
$f_{04}$	1.222	$f_{15}$	−139.413	$f_{26}$	−1.762
$f_{05}$	2.486 × 10^−1^	$f_{16}$	17.694	$f_{27}$	−1.129
$f_{06}$	62.361	$f_{17}$	1.106	$f_{28}$	1.041
$f_{07}$	−7.463	$f_{18}$	1.913 × 10^−1^	$f_{29}$	4.270 × 10^−3^
$f_{08}$	1.443 × 10^−1^	$f_{19}$	−1.521		
$f_{09}$	−1.890 × 10^−2^	$f_{20}$	2.146		
$f_{10}$	1.327333	$f_{21}$	1.616		
